# Expression of the pair-rule gene homologs *runt*, *Pax3/7, even-skipped-1 *and *even-skipped-2 *during larval and juvenile development of the polychaete annelid *Capitella teleta *does not support a role in segmentation

**DOI:** 10.1186/2041-9139-3-8

**Published:** 2012-04-18

**Authors:** Elaine C Seaver, Emi Yamaguchi, Gemma S Richards, Néva P Meyer

**Affiliations:** 1 Kewalo Marine Laboratory, Pacific Biosciences Research Center, University of Hawaii, 41 Ahui Street, Honolulu, HI, USA; 2 Sars International Centre for Marine Molecular Biology, Bergen, Norway; 3 Biology Department, Clark University, Worcester, MA, USA

**Keywords:** *Capitella*, *Eve*, *Pax3/7*, polychaete, *Runt*, Segmentation

## Abstract

**Background:**

Annelids and arthropods each possess a segmented body. Whether this similarity represents an evolutionary convergence or inheritance from a common segmented ancestor is the subject of ongoing investigation.

**Methods:**

To investigate whether annelids and arthropods share molecular components that control segmentation, we isolated orthologs of the *Drosophila melanogaster *pair-rule genes, *runt*, *paired *(*Pax3/7*) and *eve*, from the polychaete annelid *Capitella teleta *and used whole mount *in situ *hybridization to characterize their expression patterns.

**Results:**

When segments first appear, expression of the single *C. teleta runt *ortholog is only detected in the brain. Later, *Ct-runt *is expressed in the ventral nerve cord, foregut and hindgut. Analysis of *Pax *genes in the *C. teleta *genome reveals the presence of a single *Pax3/7 *ortholog. *Ct-Pax3/7 *is initially detected in the mid-body prior to segmentation, but is restricted to two longitudinal bands in the ventral ectoderm. Each of the two *C. teleta eve *orthologs has a unique and complex expression pattern, although there is partial overlap in several tissues. Prior to and during segment formation, *Ct-eve1 *and *Ct-eve2 *are both expressed in the bilaterial pair of mesoteloblasts, while *Ct-eve1 *is expressed in the descendant mesodermal band cells. At later stages, *Ct-eve2 *is expressed in the central and peripheral nervous system, and in mesoderm along the dorsal midline. In late stage larvae and adults, *Ct-eve1 *and *Ct-eve2 *are expressed in the posterior growth zone.

**Conclusions:**

*C. teleta eve, Pax3/7 *and *runt *homologs all have distinct expression patterns and share expression domains with homologs from other bilaterians. None of the pair-rule orthologs examined in *C. teleta *exhibit segmental or pair-rule stripes of expression in the ectoderm or mesoderm, consistent with an independent origin of segmentation between annelids and arthropods.

## Background

The evolution of segmentation in bilaterian animals is an ongoing area of investigation and debate (reviewed in [1-4]). Three major animal clades contain segmented representatives, the Annelida, the Arthropoda and the Chordata. According to molecular phylogenies [5,6], each of these clades is more closely related to animals that lack a segmented body than to each other. Thus, the question remains whether segmentation arose independently in distinct lineages, or whether most extant clades lost the segmented body plan that was present in a common segmented ancestor. One approach to address this question is to compare the molecular mechanisms controlling segment generation across taxa, with the assumption that shared molecular mechanisms reflect a common evolutionary history.

Segment formation is best understood in *Drosophila melanogaster*, in which a detailed understanding of the genetic control of segmentation has served as a framework for comparative studies in other arthropods. Briefly, a segmentation gene cascade sequentially divides the embryo into smaller units through the hierarchical action of the gap, pair-rule and segment polarity genes (reviewed in [7]). The pair-rule genes are the first to be expressed in a periodic pattern in *D. melanogaster*, and include the genes *paired *(*Pax3/7*), *even-skipped *(*eve*) and *runt*. These genes are expressed with a two-segment periodicity in ectodermal stripes along the anterior-posterior axis. Mutants of pair-rule genes lack alternating segmental structures such as denticle bands in the larval cuticle [8]. In contrast, mutants in segment polarity genes exhibit defects in the pattern of every segment.

The mode of segmentation in *D. melanogaster *is derived compared to other arthropods (reviewed in [9]). In *D. melanogaster*, all segments form at virtually the same time (long germ band development) and much of the patterning critical for segment formation occurs prior to cellularization. In most other arthropods, segments form in a temporal progression from anterior to posterior and the earliest segments form before the tissue for additional segments is present (short germ band development). Although many insects also develop via an early syncytial stage, this is a derived feature within arthropods, and many arthropods form all or most segments within a cellular environment.

The expression patterns of pair-rule and segment polarity gene orthologs have been examined across arthropods, including in insects, and also in chelicerates, myriapods and crustaceans. In general, pair-rule genes show more variability in their expression patterns than do the highly conserved segment polarity genes, and there are examples lacking periodic expression patterns even within insects [10]. In several arthropods, pair-rule gene orthologs are expressed in stripes in every segment; in some cases the onset of expression is clearly prior to segment generation. This pattern is more consistent with a segment polarity role than a pair-rule function. For example, in the centipede *Lithobius atkinsoni *[11] and the spider *Cupiennius salei *[12], *Pax3/7 *is expressed in a portion of every segment. In the spider mite *Tetranychus urticae *[13] and the millipede *Glomeris marginata *[14], *runt *has a segmental pattern. Likewise, *eve *is expressed with a segmental periodicity in *L. atkinsoni *[15], *C. salei *[16] and in the insect *Oncopeltus fasciatus *[17]. In *D. melanogaster *it is notable that several pair-rule genes, including *even-skipped*, *runt *and *paired*, have an initial pair-rule expression pattern of seven alternating stripes that later matures into a segmental 14-stripe pattern [18-20]. These data suggest that the pair-rule orthologs have a general, and likely ancestral, function in arthropod segment formation. The extent to which pair-rule patterning is conserved across arthropods, and whether pair-rule patterning was utilized primarily in holometabolous insects (flies, bees, beetles and moths) or ancestrally at the base of arthropods, remains an open question [7,11,18]. Regardless, the pair-rule genes are useful markers for inter-taxonomic comparisons to reconstruct the evolution of segmentation.

The mechanistic understanding of the molecular control of segment formation in annelids is poor compared to that in chordates and arthropods. Efforts to identify genes involved in annelid segmentation have largely utilized a candidate gene approach. Examining the expression of genes involved in arthropod segmentation and vertebrate somitogenesis and identification of shared components of a common genetic program for segment formation would support a shared evolutionary origin of segmentation. Expression patterns of segment polarity gene orthologs have been examined in a number of annelid species; the most well-characterized is the segment polarity gene *engrailed*. In contrast to the highly conserved expression pattern of *en *across arthropods [15,21-23], *en *expression patterns among annelids exhibit substantial variability. Although *en *is expressed in ectodermal stripes in the polychaete annelid *Platynereis dumerilii *[24], this pattern is not apparent in any other annelid examined, including in the polychaetes *Chaetopterus *sp. [25], *Hydroides elegans *and *Capitella teleta *[26] Blake, Grassle & Eckelbarger, 2009 [27] (previously known as *Capitella *sp. I) and in the leech *Helobdella triserialis *[28]. Thus, there is currently a discrepancy in *en *expression patterns among annelids and the *P. dumerilii *pattern may represent a convergence, rather than a common origin, with the arthropod pattern. Alternatively, if the *P. dumerilii *and arthropod patterns represent a conservation, there has been extensive divergence in *en *expression among annelids.

Previously, we investigated the evolution of bilaterian segmentation by characterizing the expression of orthologs of the segment polarity genes, *en*, *wg *and *hh *[26] and of the pair-rule genes *hairy *[29] and *odd-paired *[30] in *C. teleta*. Here, we report the expression of the pair-rule gene orthologs *runt*, *paired *(also called *pax group III *or *Pax3/7*) and two *eve *genes in *C. teleta *immediately prior to and during larval segment formation. Additionally, we characterize *Pax3/7 *and *eve *expression during adult segment formation. *C. teleta eve, Pax3/7 *and *runt *genes lack segmental or pair-rule stripes of expression in both the ectoderm and mesoderm, even though *Ct-Pax3/7*, *Ct*-*eve1 *and *Ct*-*eve2 *expression is initiated prior to overt segmentation. Each ortholog examined has a distinct expression pattern and exhibits expression domains conserved with those found in other bilaterians.

## Methods

### Animal husbandry

A *C. teleta *colony was maintained in the laboratory according to published culturing methods [31]. Embryos and larvae were recovered as previously described [32].

### Cloning and sequencing of *C. teleta runt*, *Pax3/7*, and *eve *orthologs

Fragments corresponding to conserved regions of *runt*, *Pax3/7 *and *eve *orthologs were isolated from *C. teleta *by degenerate polymerase chain reaction (PCR) using a cDNA template prepared from mixed embryonic and larval stages. To recover a fragment of *runt*, two rounds of amplification were performed in a semi-nested PCR reaction using the following primers from published sequences [33]: runfw-1: 5'- RCNRYNATGAARAAYCARGTNGC -3' and runbw: 5'-CKNGGYTCNCKNGGNCCRTC-3' followed by runfw-2: 5'-MRNTTYAAYGAYYTNMGNTTYGTNGG -3' and runbw. To isolate a fragment of *Pax3/7 *from mixed stage cDNA, a semi-nested approach was utilized with the following published primers [34] representing the conserved paired domain and paired-like homeodomain: Prbyfout: 5'- GGNGGNGTNTTYATHAAYGG -3' and Prbyr: 5'-RTTNSWRAACCANACYTG -3' followed by Prbyfin: 5'-MARATHGTNGARATGGC -3' and Prbyr in a second round of amplification. A 226 base pair (bp) fragment of *eve *was recovered from a genomic DNA template using the following degenerate primers: ab-evefw: 5'-MGTTAYMGTACIGCITTYAC-3' and eve-bw1: 5'-CKYTGNCKYTTRTCYTTCAT-3' [33]. Additional sequences for *Ct-eve*, *Ct-Pax3/7 *and *Ct-runt *were obtained from a mixed stage embryonic and larval stage template using gene-specific primers with the Smart rapid amplification of cDNA ends (RACE) amplification kit (Clontech, Mountain View, CA, USA). Resulting fragments were cloned into the pGEM-T Easy vector (Promega, Fitchburg, WI, USA) and sequenced by the University of Hawaii sequencing facility (Honolulu, HI, USA) or by Macrogen Inc. (Seoul, South Korea). The resulting products were: a 702 bp 5' RACE fragment and a 1269 bp 3' RACE fragment for *Ct-Pax3/7*, a 644 bp 5' RACE fragment and a 945 bp 3' RACE fragment for *Ct-runt*. For *eve*, a 460 bp 5' RACE fragment was recovered by PCR using gene-specific primers. Sequencing of multiple cDNA clones from 5' RACE reactions revealed the presence of a five amino acid insertion in some clones. Two distinct 3' RACE fragments were recovered: a 583 bp and a 734 bp fragment. Gene-specific primers designed to each of the 5' and 3' *eve *sequence variants were used to amplify and characterize features of individual cDNAs. Subsequent to the isolation of *Ct-runt*, *Ct-Pax3/7*, *Ct-eve1 *and *Ct-eve2 *by degenerate PCR, sequences became available for the *C. teleta *genome project (Department of Energy, Joint Genome Institute, Walnut Creek, CA, USA [35]). Cloned sequences were consistent with predicted gene models from the genome. The genome was searched for additional *runt*, *Pax3/7 *and *eve *paralogs; none were detected. Linkage analysis of *Ct-eve1 *and *Ct-eve2 *genes was performed through searches of the *C. teleta *genome using nucleotide sequences obtained from degenerate and RACE PCR clones. Accession numbers for *Ct-runt*, *Ct-eve1*, *Ct-eve2 *and *Ct-Pax3/7 *are listed in Additional file 1: Table S1.

### Sequence alignments and phylogenetic analyses

Sequences isolated by degenerate PCR and RACE were assigned putative orthologies based on BLASTX searches of the GenBank database from the National Center for Biotechnology Information. In addition, tBLASTn searches of the *C. teleta *genome (Joint Genome Institute (JGI), Walnut Creek, CA, USA) were conducted to find all homologs of *eve*, *runt *and the *paired *homeobox (*pax*) family. Two putative orthologs were found for *eve*, one for *runt *and six for *pax *family members. Amino acid sequences from diverse animal taxa were downloaded from the protein database in GenBank. Alignments of conserved domains were generated with ClustalX, using default parameters in MacVector 11.1.1 (MacVector, Inc., Cary, NC, USA). The domains analyzed were the homeodomain and 10 amino acids flanking the 5'- and 3'-ends of the homeodomain for Eve, the runt domain for Runt and the 127 amino acid paired domain for the Pax family. Alignments were edited by hand to correct obvious alignment errors. Nexus alignments are available upon request. Sequences, abbreviations and accession numbers used are included in Additional file 1: Table S1.

ProtTest v2.4 [36] was run for each alignment to determine the appropriate model of protein evolution. The Jones model was recommended and used for the Eve and the Pax family, and the RtRev model for Runt. Both Bayesian and maximum likelihood methods were used for each gene family. Bayesian analysis was conducted using MrBayes v3.1.2 [37], with the model determined by ProtTest. For the Runt and Pax alignments, three million generations were run, sampled every 100 generations, with four independent runs and four chains. Ten million generations were run for the Eve alignment. Once convergence was reached, majority rule consensus trees were generated with burnin values of 25,000 (Eve), 9,700 (Runt) and 5,400 (Pax). Maximum likelihood analyses were performed with RAxML v7.0.0 [38] using the same models as for Bayesian analysis with 1,000 bootstrap replicates. Trees were visualized with FigTree v1.3.1 [39] and drawn using Adobe Illustrator version CS4.

### Whole mount *in situ *hybridization

Whole mount *in situ *hybridization was performed according to previously published protocols [26]. Digoxigenin-labeled riboprobes (dig-11-UTP; Roche Diagnostics, Indianapolis, IN, USA) were transcribed with the MEGAscript High Yield Transcription Kit (Ambion, Austin, TX, USA) and diluted to working concentrations of 3 ng/μL for *Ct-runt*, 0.25 ng/μL for *Ct-Pax3/7*, 0.1 ng/μL for *Ct-eve1 *and 0.2 ng/μL for *Ct-eve2*. Probe lengths were as follows: 1041 bp for *Ct-runt*, 1270 bp for *Ct-Pax3/7*, 1379 bp for *Ct-eve1 *and 1224 bp for *Ct-eve2*. Following termination of the development reaction, specimens were equilibrated and stored in 80% glycerol, 20% 5× phosphate buffered saline (PBS). The detailed protocol is available upon request.

### Visualization of mid-body mesoderm

Animals were fixed and labeled according to previously described conditions (fixation #4, [40]). Specimens were exposed to a 1:500 dilution of mouse anti-histone antibody (F152, C25.WJJ; Millipore, Billerica, MA, USA) and a 1:400 dilution of goat anti-mouse rhodamine (Invitrogen, Carlsbad, CA, USA). After antibody labeling, animals were incubated in 1:100 BODIPY FL phallacidin (Invitrogen) in PBS, 0.1% Triton X-100 (PBT) for two hours at room temperature followed by several PBT washes over 45 minutes. Animals were mounted in Slow Fade Gold (Invitrogen) and analyzed.

### Microscopy

Riboprobe-labeled specimens were analyzed using differential interference contrast (DIC) optics on an Axioskop 2 compound microscope (Zeiss, Munich, Germany). Digital images were captured with either a stem-mounted 4.0 megapixel Nikon Coolpix 4500 (Nikon, Inc., Melville, NY, USA) or SpotFlex digital camera (Diagnostic Instruments, Inc., Sterling Heights, MI, USA). Confocal laser scanning microscopy was performed using an Axioplan 2 LSM510 microscope (Zeiss). Three-dimensional reconstructions were generated from confocal images with ImageJ (National Institutes of Health, Bethesda, MD, USA). Figures and diagrams were constructed using Photoshop CS4 and Illustrator CS4 (Adobe Systems Inc., New York, NY, USA). Some panels in figures seven and eight (see Figure legends) are composites of multiple DIC focal planes that were merged using Helicon Focus (Helicon Soft Ltd., Kharkov, Ukraine).

## Results

### Cloning and phylogenetic analyses of *C. teleta runt*, *eve *and *Pax3/7 *genes

**Figure 1:**
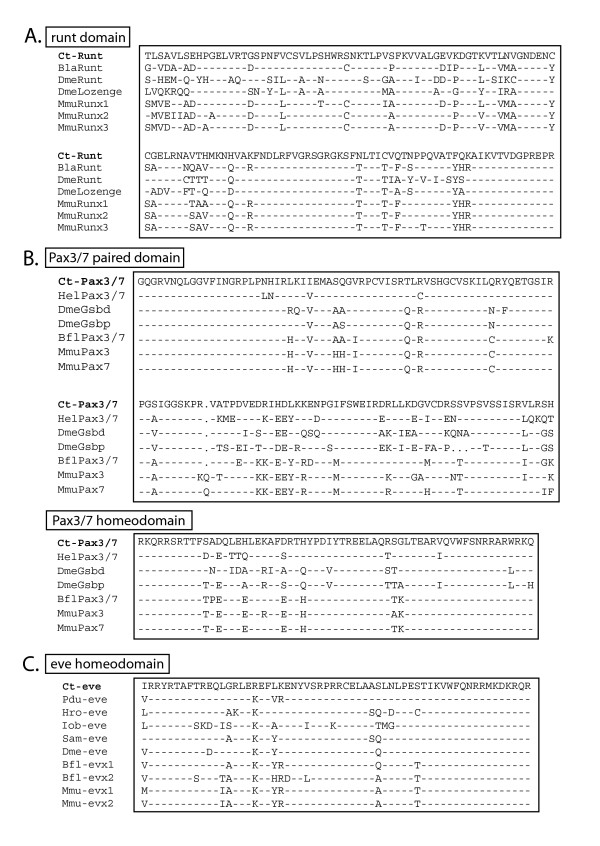
**Alignments of conserved domains in Ct-Runt, Ct-Pax3/7 and Ct-Eve**. **(A) **Amino acid alignment of the Runt domain of Ct-Runt compared to the Runt domain of other animals. **(B) **Amino acid alignment of Ct-Pax3/7 and Pax3/7 proteins from other species. The alignments shown include the paired domain and the homeodomain. **(C) **Amino acid alignment of the Ct-Eve homeodomain with homeodomains from Eve proteins in a range of species. Dashes represent amino acid identities with the *C. teleta *sequences; dots represent gaps introduced by CLUSTALW to optimize alignments. Bfl: *Branchiostoma floridae*; Bla: *B. lanceolatum*; Dme: *Drosophila melanogaster*; Hel: *Helobdella *sp.; Hro: *H. robusta*; Mmu: *Mus musculus*; Pdu: *Platynereis dumerilii*; Iob: *Ilyanassa obsoleta*; Sam: *Schistocerca Americana*.

Using degenerate and RACE PCR, we isolated fragments comprising 1585 bp of a *C. teleta runt *ortholog, designated *Ct-runt*. The sequence contained a 157 bp 5' untranslated region (UTR), a 570 bp open-reading frame (ORF) predicting a protein of 190 amino acids and an 857 bp 3' UTR and poly-A tail. The predicted ORF included a conserved Runt DNA binding domain of 128 amino acids. When compared to Runt sequences from other organisms, the *Ct-runt *predicted ORF showed strong conservation of amino acid residues throughout the Runt DNA binding domain (Figure 1A). Phylogenetic analyses of the Runt domain from Ct-Runt were performed using both Bayesian and maximum likelihood methods. Ct-Runt groups most closely with the Lozenge protein from the parasitic flatworm *Schistosoma mansoni*, another lophotrochozoan, and these two sequences fall within a group that contains ecdysozoan, lophotrochozoan and non-vertebrate deuterostome Runt sequences (Additional file 2: Figure S1). The vertebrate and cephalochordate Runt and Runx sequences form a well-supported separate group, and cnidarian sequences fall outside of both groups.

The *C. teleta runt *ortholog we recovered lacks the characteristic motif VWRPY at the C-terminal end, which is necessary for Groucho-mediated transcriptional repression activity [41]. To confirm that the missing motif was not due to a cloning artifact, 3' RACE PCR was repeated with a second primer set and an independently generated cDNA pool. The resulting fragment was identical in sequence to the original *Ct-runt *3' RACE product. In addition, we identified a single *runt *sequence from searches of the *C. teleta *genome [35], which has the same amino acid sequence as the sequence isolated by PCR and lacks the C-terminal Groucho-binding domain. Searches of the *Lottia gigantea *genome [42] reveal that its predicted *runt *homolog also lacks the VWRPY C-terminal motif (Protein ID 119594).

**Figure 2:**
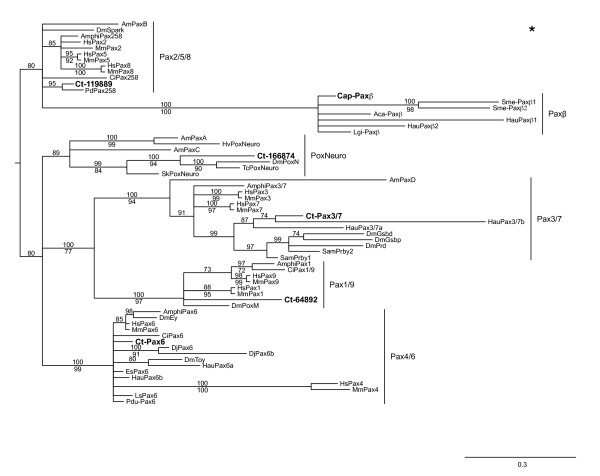
**The genome of *Capitella teleta *has six members of the Pax family of proteins**. *C. teleta *Pax family members group with the following: Pox neuro, Paxβ, Pax1/9, Pax2/5/8, Pax3/7, and Pax4/6. The tree shown is a Bayesian consensus tree, with posterior probability support values placed above the nodes. Maximum likelihood bootstrap support values are included where the tree topology agreed with the Bayesian consensus tree, and are below the nodes. Am: *Acropora millepora*; Amphi: *Branchiostoma floridae*; Ci: *Ciona intestinalis*; Ct-: *Capitella teleta*; Dj: *Dugesia japonica*; Dm: *Drosophila melanogaster*; Es: *Euprymna scolopes*; Hau: *Helobdella *sp. Austin; Hs: *Homo sapiens*; Ls: *Lineus sanguineus*; Mm: *Mus musculus*; Pdu-: *Platynereis dumerilii*; Sam: *Schistocerca Americana*.

A paired (*Pax3/7*) ortholog, *Ct-Pax3/7*, was amplified by degenerate and RACE PCR from a mixed stage cDNA template. This sequence was composed of a 99 bp 5' UTR, a 1536 bp ORF predicting a protein of 416 amino acids and a 175 bp 3' UTR that includes a poly-A tail. Pax3/7 proteins are defined by the presence of three conserved domains: a paired domain and 'paired' class homeodomain, both of which are DNA binding domains, and a short octapeptide motif, which is typically located in the linker region between the paired domain and the homeodomain. This octapeptide motif has been shown to act as a site for Groucho-mediated repression [43]. The predicted *Ct-Pax3/7 *ORF from the *C. teleta *cDNA contains a putative paired domain and a 'paired' class homeodomain (Figure 1B) but lacks the octapeptide motif in the linker region. To confirm our placement of *Ct-Pax3/7 *within the Pax3/7 family, we identified other putative *Pax *homologs in the *C. teleta *genome and ran phylogenetic analyses using the paired domain. Several distinct Pax families are broadly distributed across bilaterians and include: Pax1/9, Pax2/5/8, Pax3/7, Pax4/6 and Pox neuro [44]. More recently, a Paxβ family has been described, a novel Pax subfamily only found in lophotrochozoans [45]. *C. teleta *has six Pax family members, with a single representative in each of the Pax families Pox neuro, Paxβ, Pax1/9, Pax2/5/8, Pax3/7 and Pax4/6 (Figure 2). Both Bayesian posterior probability and maximum likelihood bootstrap show strong support for the distinct Pax gene groups. Ct-Pax3/7 groups most closely with the two Pax3/7 sequences from another annelid, the leech *Helobdella *sp. Austin. These lophotrochozoan Pax3/7 sequences group separately from the deuterostome Pax3 and Pax7 clade and the arthropod homologs Gooseberry, Pairberry and Paired.

A 226 bp fragment of *eve *was initially identified by degenerate PCR from a *C. teleta *genomic DNA template. Subsequent RACE PCR recovered a 5' fragment and two distinct 3' fragments. From sequence searches of the *C. teleta *genome, two distinct *eve *genes were identified, which we designate *Ct-eve1 *and *Ct-eve2*. The *Ct-eve1 *and *eve2 *predicted ORFs are 325 and 330 amino acids, respectively. The Ct-Eve1 and Ct-Eve2 predicted ORFs are largely identical within the predicted ORF; the exceptions are the two amino acids immediately 5' of the stop codon (KS for *Ct-eve1 *and PK for *Ct-eve2*), and a small five amino acid insertion present only in Ct-Eve2. This insertion immediately follows amino acid residue 85 and is located 17 amino acids 5' of the homeodomain. The homeodomain in both Eve sequences contains conserved amino acids characteristic of *eve *gene sequences (Figure 1C). Bayesian and maximum likelihood analyses of the homeodomain confirm the identity of *Ct-eve1 *and *Ct-eve2 *as *eve *gene orthologs within the homeodomain superfamily (Additional file 3: Figure S2). Ct-Eve1 and Ct-Eve2 cluster together with Eve proteins from other animal taxa and separately from the homeodomain-containing Gbx and Engrailed outgroups. There is 100% Bayesian posterior probability and 90% bootstrap support for the Eve node. Within the predicted ORFs, there are only seven nucleotide differences between *Ct-eve1 *and *Ct-eve2*, aside from sequence differences due to the five amino acid insertion in Ct-Eve2 and the two amino acids immediately 5' of the stop codon. In contrast, the two sequences diverge in sequence and length in the 3' UTR (*Ct-eve1*, 167 bp and *Ct-eve2*, 349 bp).

**Figure 3:**
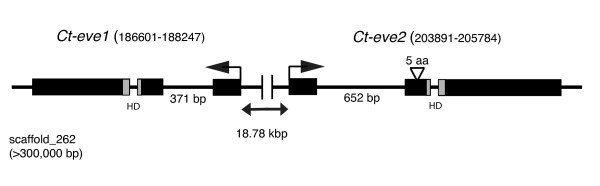
**Genomic organization of *eve *genes in *Capitella teleta***. The two *C. teleta eve *genes are positioned 18.78 kbp apart on the same scaffold. Boxes indicate exons and lines between the boxes indicate introns. The sizes of intron 1 for both genes are indicated beneath the intron. Arrows indicate the direction of transcription. The five amino acid insertion 5' of the homeodomain in *Ct-eve2 *is marked. Gene names are indicated with scaffold coordinates in parentheses. The scaffold number and scaffold length is written at the left of the schematic. HD: homeodomain.

Both *Ct-eve1 *and *Ct-eve2 *are located on scaffold 262, approximately 18.7 kbp apart, and these two genes exhibit opposite transcriptional orientations (Figure 3). For both genes, the predicted ORFs are distributed among three exons, and the positions of the intron-exon boundaries are shared between the two genes. In contrast, the size and sequence composition of the first intron are different between *Ct-eve1 *(371 bp) and *Ct-eve2 *(652 bp). The second intron is the same in length and sequence in the two genes and is located within the homeodomain, between homeodomain positions 46 and 47. The position of the second intron within the homeodomain is conserved in the two amphioxus and mouse *eve *gene homologs and the single *Tribolium castaneum eve *gene [46,47], and is different from the position of the single intron in the *D. melanogaster eve *gene, which is upstream of the homeodomain [48]. Exon 3 for both genes also contains 191 amino acids of the ORFs, as well as the gene-specific 3' UTRs. Comparisons of the 3 kbp of 5' and 3' sequence flanking the two ORFs lack regions of sequence similarity between *Ct-eve1 *and *Ct-eve2*. The *C. teleta eve *genes are not clustered with the *Hox *genes in the genome [49], although it remains a possibility that they are all on the same chromosome. In *P. dumerilii*, *eve *is on the same chromosome as the posterior class Hox gene *Post2*, but is not part of the Hox cluster [50], and *T. castaneum eve *is also not clustered with the Hox genes [47]. In contrast, *eve *orthologs in vertebrates and amphioxus, and in the cnidarian *Nematostella vectensis*, are clustered with the Hox genes [51-53].

### *C. teleta *development

**Figure 4:**
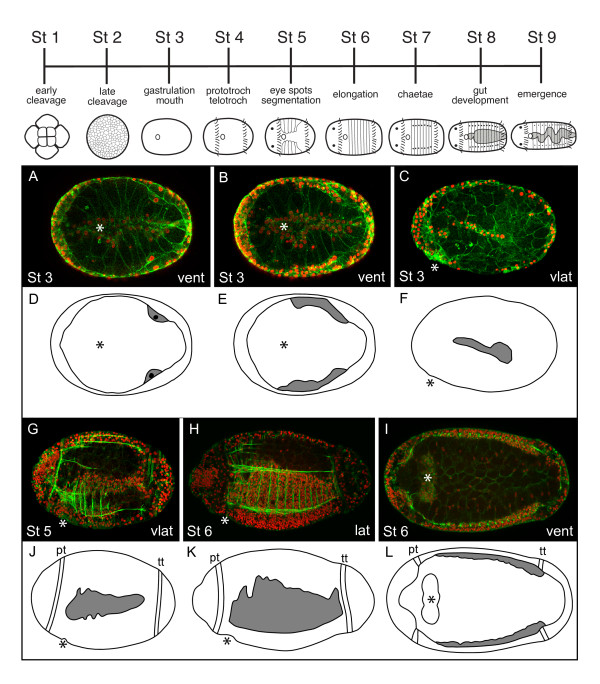
**Expansion of mesodermal bands in *Capitella teleta***. Stages are marked in the lower left corner of each panel. Anterior is to the left for all panels. Images in A, B, D, E, I and L are in a ventral (vent) orientation;images in C, F, G, H, J and K are in a ventrolateral (vlat) or lateral (lat) orientation. The approximate position of the mouth (asterisk) is indicated. At the top is a developmental staging chart for *C. teleta*. **(A-C,G-I) **Confocal z-stack projections through embryos (Stage 3) and larvae (Stage 5, Stage 6) labeled with BODIPY FL phallacidin (green; cell outlines and muscle fibers) and anti-Histone antibody (red; nuclei). **(D-F,J-L) **Each diagram is drawn from the confocal z-stack projection directly above it. The pair of large posterior mesodermal cells (D) or the mesodermal bands (E, F, J-L) are shown in grey. lat: lateral; pt: prototroch; tt: telotroch vent: ventral; vlat: ventrolateral.

*C. teleta *development has been described previously and a standard staging system is used to distinguish among embryonic and larval stages (Figure 4 top) [32]. Following unequal spiral cleavage (Stages 1 to 2) and gastrulation (Stage 3), two trochal bands, the prototroch and telotroch, penetrate through the egg membrane to initiate the larval phase (Stages 4 to 9). The prototroch and telotroch delineate, respectively, the anterior and posterior boundaries of the segmented mid-body. The mouth is positioned immediately posterior of the prototroch, on the ventral side of the larva. The first four to five segments initially appear during early larval stages (Stage 5), and segmental furrows are visible on the ventral face of the larva [28,32]. The first ten segments form progressively from anterior to posterior within a 24 hour time frame, and three additional larval segments form from a posterior growth zone (PGZ) by late larval stages (Stage 9). The final three larval segments form at a rate of approximately one per day. Following metamorphosis, 40 to 50 additional posterior segments are generated from the PGZ.

The segmented ectoderm and mesoderm have distinct embryonic origins in *C. teleta *[54]. All segmental ectoderm is generated from the blastomere 2d, which also generates nonsegmental posterior ectoderm. The segmented mid-body ectoderm segregates from nonsegmental ectoderm within the first few divisions of 2d, such that 2d^11 ^generates segmented ectoderm and PGZ, but not the telotroch or the posterior unsegmented pygidium [54]. Most of the mid-body mesoderm arises from the two cells 3c and 3d, whose descendants form the right and left mesodermal bands, respectively (Figure 4). Initially, a pair of large posterior mesodermal cells is visible beneath the ectoderm (Figure 4A,D). Subsequently, two mesodermal bands become visible as a row of subsurface cells in the lateral part of the body (Figure 4B,C,E,F). Over time, the bands increase in cell number and expand circumferentially (Figure 4G-L). By stage 5, when the first segments are visible, longitudinal and circular muscle fibers extend from the mesodermal bands (Figure 4G). Extension of a single circular muscle fiber per segment is one of the earliest signs of segmentation in the mesoderm and follows soon after ectodermal segmentation. These circular muscle fibers are positioned within the intersegmental grooves (Figure 4I) [32].

### *Ct-runt *expression

**Figure 5:**
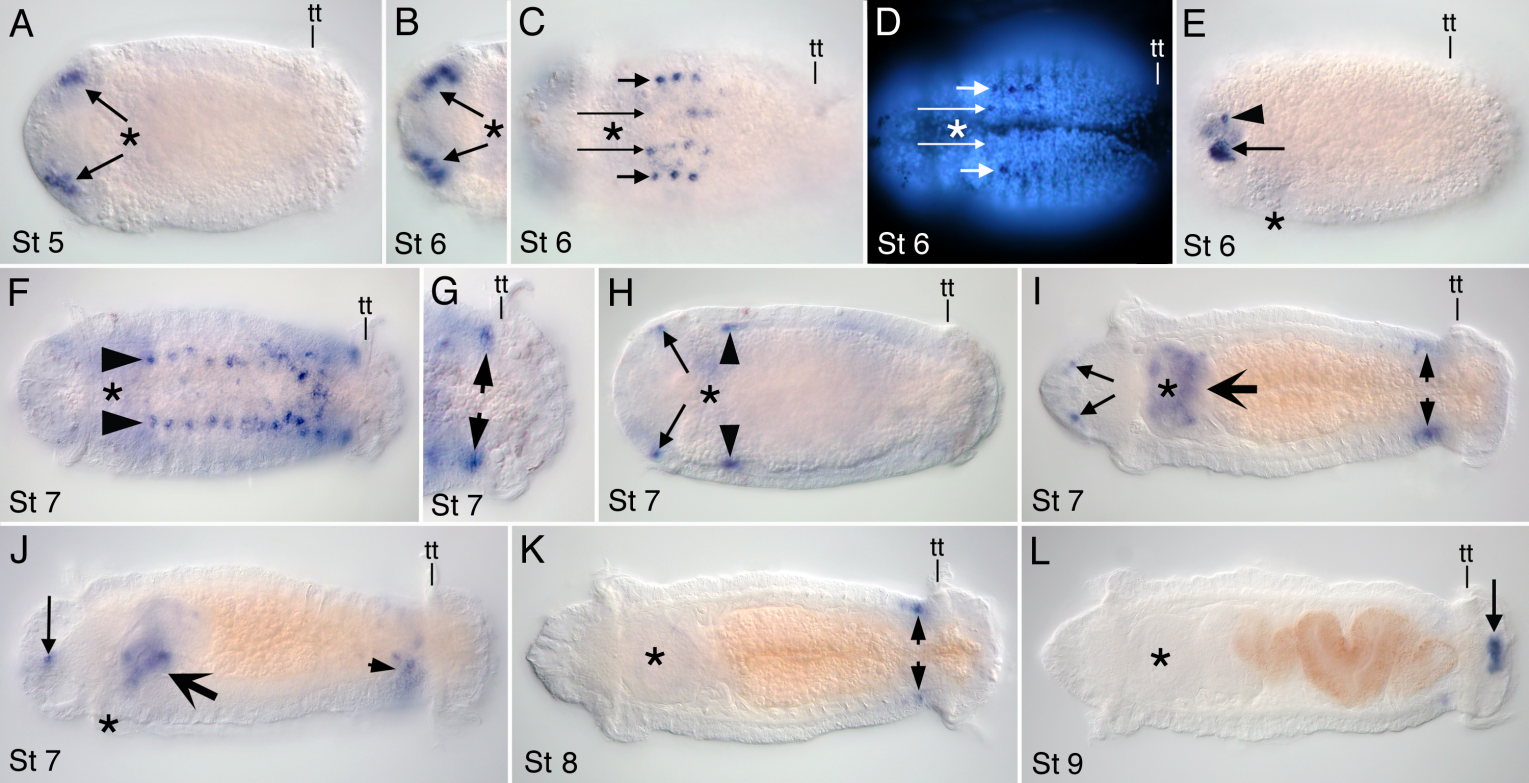
**Larval expression of *Ct-runt***. Stages are marked in the lower left corner of each panel. Anterior is to the left for all panels. All panels are ventral views except for E and J, which are lateral views with ventral down. An asterisk marks the position of the mouth. **(A) ***Ct-runt *expression in the brain (arrows). **(B,C) **Two different focal planes of the same animal showing expression in the brain (B) and ventral nerve cord in anterior segments (long arrows in C). Short arrows point to expression in ventrolateral ectodermal cells. **(D) **Epi-fluorescence view showing position of medial (long arrows) and lateral-expressing cells (short arrows) within each segment. **(E) **Arrowhead and arrow mark discrete clusters of *Ct-runt*-expressing cells visible within a single focal plane in the brain. **(F) **Arrowheads show nervous system expression extending along the length of the ventral nerve cord. **(G) **Enlarged view of the posterior body of the larva shown in F. Arrows point to expression in the ectoderm of the PGZ. **(H) **Expression becomes restricted to a single small cluster in the brain (arrows) and in a small lateral domain of mesoderm (arrowheads). **(I) **At late Stage 7, *Ct-runt *is transiently expressed in the foregut (large arrow). There is also expression in the PGZ (short arrows) and the anterior head ectoderm (diagonal arrows). **(J) **Foregut expression is in the pharynx (large arrow). PGZ expression is ventrally localized (short arrow). Long arrow points to anterior head expression. **(K) **Expression is restricted to the PGZ at Stage 8 (short arrows). **(L) **Arrow indicates prominent expression in the hindgut of a late stage larva. tt: telotroch.

*Ct-runt *is expressed in several distinct ectodermal tissues, predominantly in the nervous system, foregut and hindgut, but is never detected in ectodermal stripes of expression in the segmented part of the body (Figure 5). Most *Ct-runt *expression is transient, lasting only one to two days. Initially, *Ct-runt *expression is localized to two small domains in the brain in Stage 5 larvae (Figure 5A), and is not detected in Stage 3 and Stage 4 larvae. The first segments appear at Stage 5, and *Ct-runt *is not expressed in nascent segments or in the presumptive segmental tissue at this stage. At Stage 6, expression in the brain appears as several discrete cell clusters (Figure 5B,E). There is also expression in the anterior four to five segments in two columns of cells (Figure 5C). The more lateral *runt*-expressing cells are centrally positioned within the segment (Figure 5D). The medial cells are clearly in the forming ventral nerve cord. Because the lateral boundary of the ventral nerve cord is not yet distinct at this stage, it is unclear if the lateral cells (short arrows) are within or lateral to the ventral nerve cord. At Stage 7, several new domains of *Ct-runt *expression appear, most notably in the foregut. At late Stage 7, discrete sub-domains of the foregut become morphologically obvious, and there is expression of *Ct-runt *in the pharynx (Figure 5J). By Stage 8, foregut expression is no longer detectable. Expression also appears in the ventrolateral ectoderm of the PGZ during Stage 7 (Figure 5G,I,J), which persists to Stage 8 (Figure 5K). At Stage 7, there is also transient expression in a small lateral mesodermal domain, posterior to the mouth (Figure 5H). In the nervous system, expression in the ventral nerve cord expands to more posterior segments and is prominent in a single row of segmentally repeated cells (Figure 5F). Brain expression ceases, although there remain *Ct-runt*-positive ectodermal cells in the head, which are likely sensory cells (Figure 5H-J). In late stage larvae, expression is limited to a ring in the ectoderm of the hindgut (Figure 5L). In some cases we also observed weak expression in the PGZ (not shown), which we interpret to be residual transcript from expression during earlier stages.

### *Ct-Pax3/7 *expression

**Figure 6:**
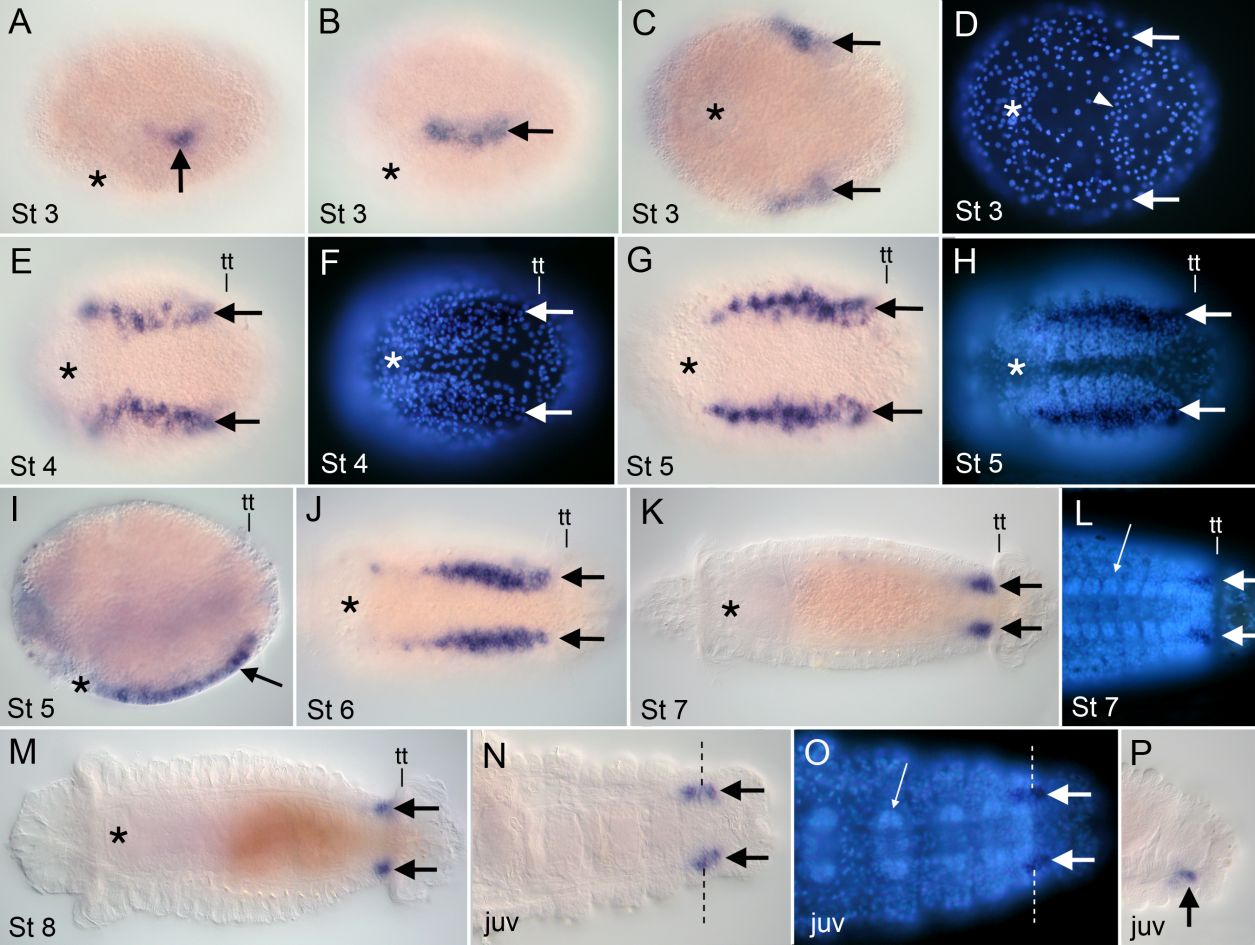
**Expression of *Ct-Pax3/7***. Stages are marked in the lower left corner of each panel. Anterior is to the left for all panels. All panels are ventral views except A, B, I and P. Panels A, B and P are lateral views with ventral down; panel I is a ventrolateral view. Specimens shown in D, F, H, L and O were stained with Hoechst. The mouth is indicated where present (asterisk). **(A) ***Ct-Pax3/7 *expression in lateral ectodermal cells (arrow) during early Stage 3. **(B,C) **By late Stage 3, expression expands into lateral bands (arrows). **(C,D) **DIC (C) and epi-fluoresence (D) images of the same animal. Arrowhead in D indicates nuclei of the presumptive telotroch. **(E,F) **DIC (E) and epi-fluorescence (F) of the same animal. By Stage 4, expression extends from the stomodeum to the telotroch (arrows). **(G,H) **DIC (G) and epi-fluorescence (H) of the same animal. Longitudinal bands of expression (arrows) persist as the first larval segments appear. **(I) **Expression in surface ectoderm (arrow). **(J) **During Stage 6, expression diminishes in anterior segments (arrows). **(K) **Expression becomes limited to the posterior segments and PGZ (arrows). **(L) **Enlarged view of same animal in K showing expression adjacent to the ganglia (large arrows). Small arrow points to ganglion of the ventral nerve cord in a more anterior segment. **(M) **At Stage 8, expression is restricted to two patches of ventrolateral ectoderm in the PGZ (arrows). **(N,O) **Posterior region of a juvenile. DIC (N) and epi-fluorescence (O) images of the same specimen showing expression in and posterior to the nascent segment (large arrows). Dotted line marks posterior boundary of the nascent segment. **(P) **Posterior end of a juvenile showing expression on the ventral side of the body (arrow). juv: juvenile; tt: telotroch.

*Ct-Pax3/7 *transcripts are detected at all developmental stages examined (Stage 3 to 9 and in juveniles) in two ventrolateral ectodermal bands oriented along the anterior-posterior axis. Following gastrulation at Stage 3, *Ct-Pax3/7 *is expressed in two small lateral domains in the posterior half of the embryo (Figure 6A). Slightly later, this expression expands anteriorly to form two bands of ectodermal expression (Figure 6B,C). This expression appears prior to the formation of the larval segments, prototroch, telotroch and the ventral nerve cord. The lateral bands of *Ct-Pax3/7 *expression continue to expand anteriorly and by Stage 4 they extend from the level of the mouth to the telotroch (Figure 6E,F). The longitudinal bands of *Ct-Pax3/7 *expression are the only detectable expression domains at every stage examined, including immediately prior to and during the appearance of the first segments (Figure 6C-H). The relative intensity of expression varies somewhat along the length of the band (Figure 6G). At Stage 6, expression in anterior segments decreases (Figure 6J), and gradually decreases from anterior to posterior. The posterior boundary of expression is stable from Stages 5 to 9; it abuts, but does not include the telotroch. Between Stage 4 and 6, the distance between the two bands of expression decreases (compare Figure 6G and 6 J), reflecting the movement of the two sides of the ventral nerve cord towards the ventral midline. By late Stage 7, *Ct-Pax3/7 *expression is limited to a small ventrolateral domain of ectoderm in the PGZ (Figure 6K). This expression is positioned immediately lateral to the ventral ganglia (Figure 6L), and persists through Stage 8 (Figure 6M). The *Ct-Pax3/7 *transcript is no longer detectable in Stage 9 larvae. In post-metamorphic juveniles, *Ct-Pax3/7 *expression is limited to a small ventrolateral domain of ectoderm that abuts the lateral edge of the ventral ganglia in the nascent segment and also extends into the growth zone (Figure 6N,O,P). Thus, in juveniles, *Ct-Pax3/7 *is expressed in a restricted area of ectoderm in the PGZ.

### Expression of *eve *genes

**Figure 7:**
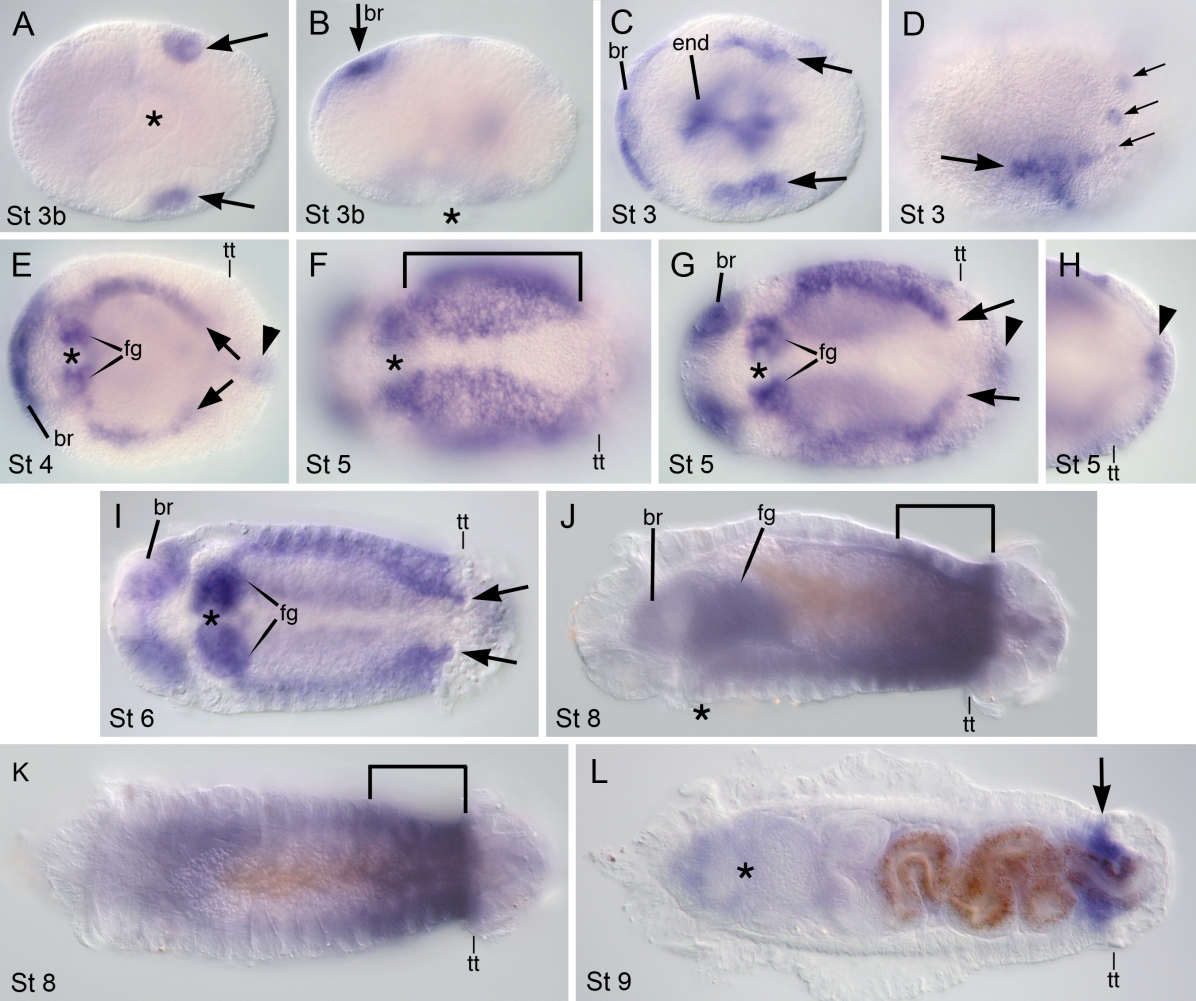
**Expression of *Ct-eve1***. Stages are marked in the lower left of each panel and anterior is to the left for all panels. Except for B, D, J (lateral views with ventral down) and K (dorsal view), all panels are ventral views. An asterisk marks the blastopore in A and B and the mouth in all other panels. **(A,B) **Ventral (A) and lateral (B) views of the same animal. At the end of gastrulation, *Ct-eve1 *is expressed in mesodermal precursor cells (arrows in A) and the presumptive brain (br). **(C,D) **Expression in the presumptive brain (br), mesodermal bands (large arrows), endoderm (end) and ectodermal cells positioned immediately anterior to the telotroch (small arrows). Panel C is a composite of multiple focal planes. **(E) **Expression is in the brain (br), presumptive foregut (fg), mesodermal bands (large arrows) and hindgut (arrowhead). **(F-H) **Expression is throughout the mid-body mesoderm as it expands circumferentially (bracket in F, arrows in G) and there is continued expression in the brain (br), foregut (fg) and hindgut (arrowhead). F-H are different focal planes of the same animal. **(I) **Expression during Stage 6 includes the brain (br), foregut (fg), mid-body mesoderm and mesodermal precursor cells (arrows). **(J,K) **By Stage 8, the most prominent expression is in the posterior mid-body (bracket), with weaker expression in the brain, foregut and hindgut. **(L) **In late larval stages, expression is largely restricted to the PGZ (arrow). br: brain; end: endoderm; fg: foregut; St 3b: Stage 3 blastopore; tt: telotroch.

**Figure 8:**
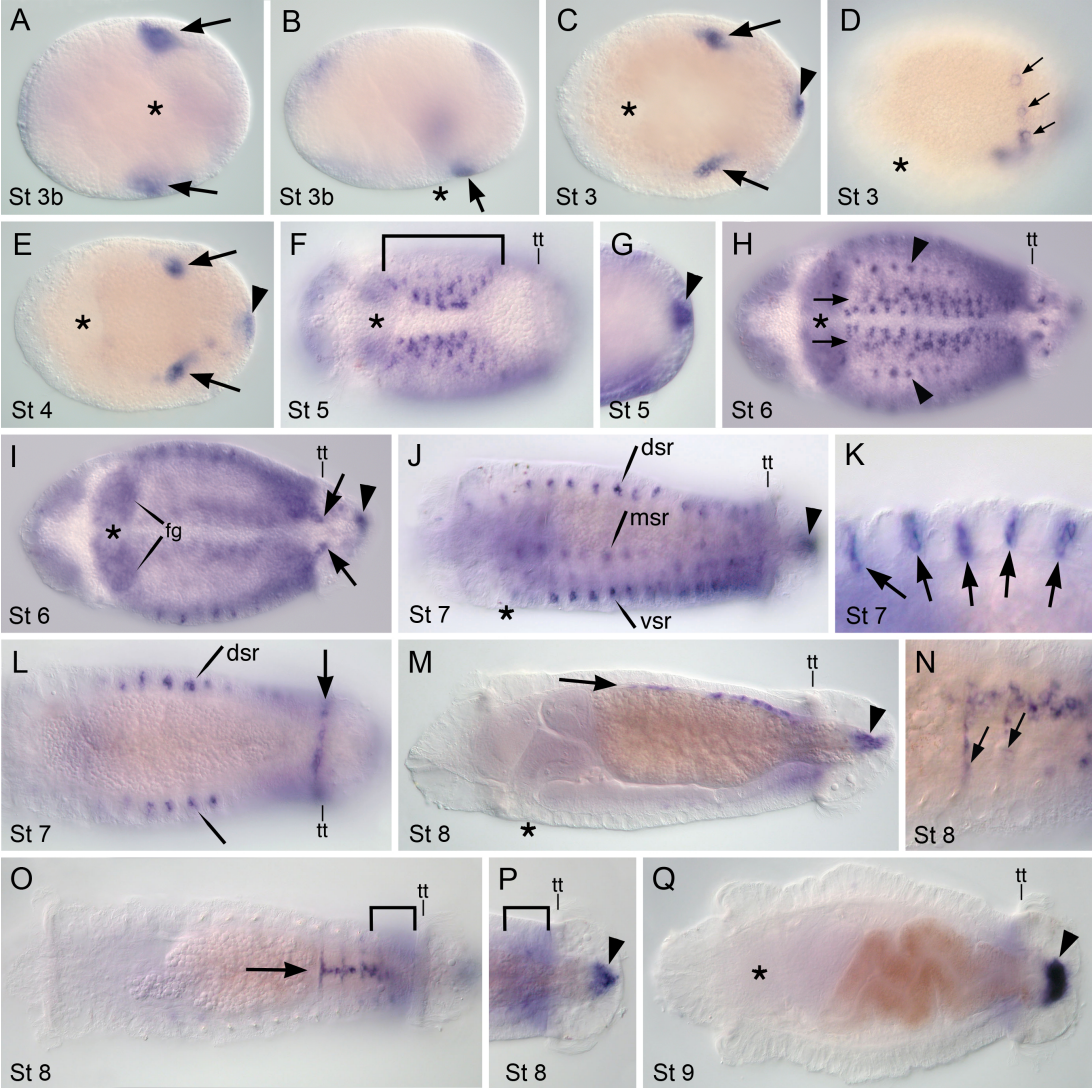
**Expression of *Ct-eve2***. Stages are marked in the lower left of each panel. Panels A, C, E, F, G, H, I and Q are ventral views; B, D, J and M are lateral views with ventral down; K is a ventrolateral view; L, O and P are dorsal views; N is a dorsolateral view. Anterior is to the left for all panels. An asterisk marks the blastopore in A and B and the mouth in all other panels. **(A,B) **Ventral (A) and lateral (B) views of the same animal. *Ct-eve2 *expression at the posterior edge of the blastopore (arrow in B) and in mesoderm precursor cells (arrow in A). **(C,D,E,G) **Expression in mesoderm precursor cells (arrow in C,E), ectodermal cells anterior to the telotroch (small arrows in D) and the hindgut (arrowhead). Panel C is a composite of multiple focal planes. **(F) **Expression in the ventral nerve cord (VNC) (bracket in F). F and G are different focal planes of the same animal. **(H) **Expression in the VNC (horizontal arrows) and ectodermal cells (arrowheads). **(I) **Expression in the foregut (fg), mid-body ectoderm, mesoderm of the PGZ (arrows) and hindgut (arrowhead). **(J-L) **Expression in three rows of ectodermal sensory structures: ventral, medial and dorsal rows. K is an enlarged view of the dorsal row of ectodermal sensory structures (arrows). Arrowhead in J points to hindgut expression. L shows the dorsal row of *Ct-eve2*-expressing cells (dsr). **(M-O) **Mesodermal expression along the dorsal midline (arrows in M,O). N is an enlarged view of dorsal mesoderm expression. Rows of labeled cells (arrows) extend from the dorsal midline. O and P are the same animal. Expression persists in the hindgut (arrowhead in M and P) and PGZ (bracket in O and P). (Q) Arrowhead shows prominent expression in the rectum in late larval stages. dsr: dorsal sensory row; fg: foregut; msr: medial sensory row; St 3b: stage 3 blastopore; tt: telotroch; vsr: ventral sensory row.

**Figure 9:**
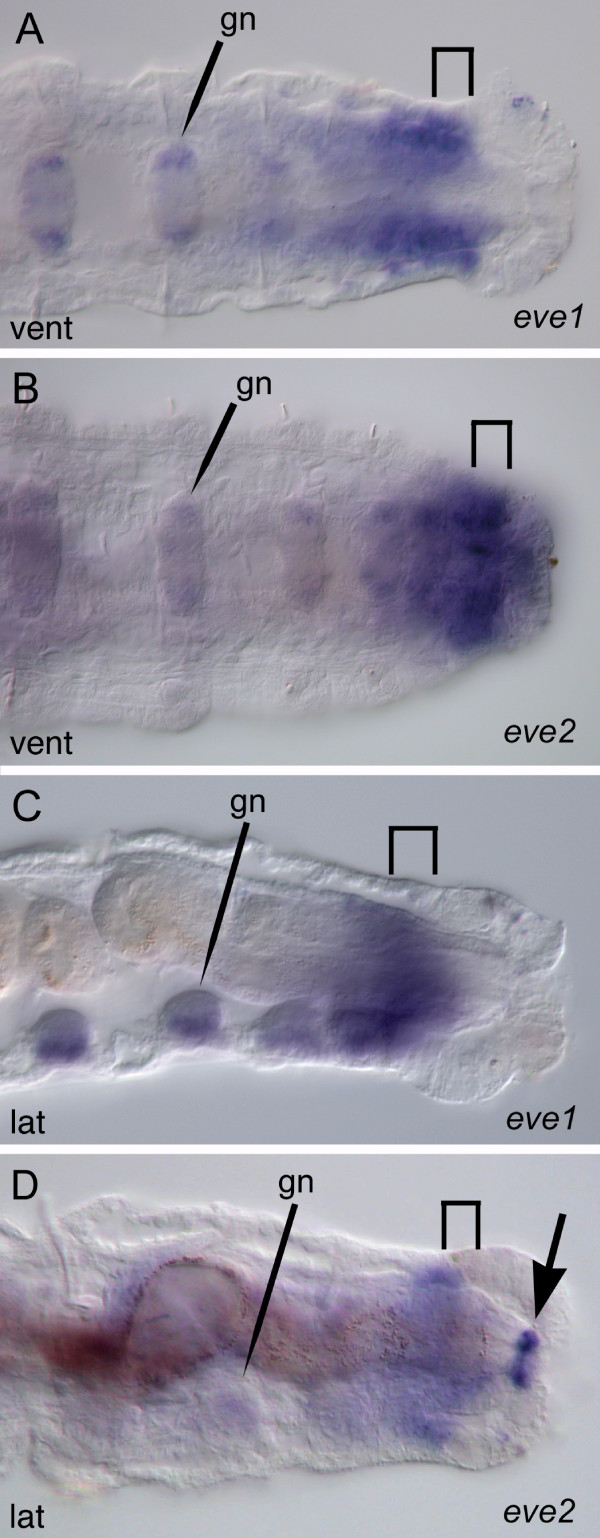
***Ct-eve1 *and *Ct-eve2 *expression in the posterior growth zone of juveniles**. All panels show the posterior end of juveniles. A and B are ventral (vent) views and C and D are lateral (lat) views with ventral down. A bracket denotes the PGZ. **(A) ***Ct-eve1 *is expressed in the ectoderm and mesoderm of the PGZ and in the ganglia of the ventral nerve cord (VNC). **(B) ***Ct-eve2 *is expressed in the PGZ and weakly in the ganglia of the VNC. **(C) ***Ct-eve1 *expression in the PGZ is more prominent on the ventral side than on the dorsal side (bracket). **(D) **In addition to PGZ expression in the ectoderm and mesoderm (bracket), *Ct-eve2 *is expressed in the anus (arrow). gn: ganglion; lat: lateral; vent: ventral.

Although the two *C. teleta eve *genes have close to 100% amino acid identity within their ORFs (see previous section), each gene has a unique expression pattern. Both genes are detected at all developmental stages examined in multiple tissues and multiple germ layers (Stages 3 to 9). However, neither *Ct-eve1 *nor *Ct-eve2 *is detected in ectodermal or mesodermal stripes in the mid-body, whether preceding or following the appearance of segments (Figures 7 and 8). *Ct-eve1 *is broadly expressed during cleavage stages (data not shown); here, we focus on spatial characterization of the *Ct-eve1 *transcript from the end of gastrulation (Stage 3) through metamorphosis (Stage 9). During gastrulation, when a blastopore is present (Stage 3b), *Ct-eve1 *is detected in two domains: in the mesodermal precursor cells (Figure 7A) and the dorsal side of the embryo, in the presumptive brain (Figure 7B). Soon after, *Ct-eve1 *is transiently expressed in the endoderm (Figure 7C). In addition, expression persists in the nascent mesodermal bands and in the forming brain (Figure 7C). During Stage 3, *Ct-eve1 *also is expressed in a fourth domain in a posterior ring of ectodermal cells that extend to the dorsal surface of the embryo (Figure 7D). Expression associated with the presumptive foregut and hindgut appears at Stage 4 (Figure 7E). The *Ct-eve1 *transcript is broadly expressed throughout the mid-body mesoderm as it expands circumferentially through Stage 5 and 6 (Figure 7F,G,I). Expression also persists in the mesodermal precursor cells, which at this stage are positioned beneath the telotroch (Figure 4C and 7I, large arrows). During this time, expression in the brain, foregut and hindgut persists from previous stages (Figure 7G,H,I). In late larval stages, there is expression in the posterior mid-body mesoderm, which gradually becomes restricted to the PGZ by Stage 9 (Figure 7J,K,L), and expression in other domains is either very weak or not detectable. In juvenile stages, there is a continuous domain of expression that includes the ectoderm and mesoderm of the PGZ and the nascent segments (Figure 9A,C). *Ct-eve1 *is also expressed in posterior ganglia of juveniles.

*Ct-eve2 *has distinct expression domains from *Ct-eve1*, although there are clear overlapping areas of expression. When the blastopore is present (Stage 3b), *Ct-eve2 *is detected in the mesodermal precursor cells (Figure 8A) and on the posterior face of the blastopore at the ventral midline (Figure 8B). Later during Stage 3, there is continued expression in the mesodermal precursor cells, and novel expression in the presumptive hindgut (Figure 8C) and in a ring of ectodermal cells near the posterior end of the embryo (Figure 8D). At Stages 4 and 5, after the telotroch has formed, it is apparent that these posterior ectodermal cells are immediately anterior to the telotroch in the position of the presumptive PGZ (not shown). *Ct-eve2 *expression in the mesodermal precursor cells and hindgut persists (Figure 8E,G). At Stage 5, *Ct-eve2 *also appears in several additional ectodermal domains, including weak expression in the foregut and mid-body ectoderm (between the prototroch and telotroch). The most prominent expression is in the forming ventral nerve cord (Figure 8F), which persists as the left and right sides fuse at the ventral midline at Stage 6 (Figure 8H). In the mid-body, there is expression in both the ectoderm and mesoderm at Stage 6. The mesodermal expression is particularly pronounced in the PGZ and in the mesodermal precursor cells (Figure 8I). In the ectoderm, small clusters of cells express *Ct-eve2 *more strongly relative to surrounding cells (Figure 8H arrowheads), which become refined to three columns of discrete, segmentally iterated cell clusters on each side of the animal by Stage 7 (Figure 8 J,K,L). The three columns are in the following positions: between the ventral nerve cord and the neuropodial chaetae, between the neuro- and notopodial chaetae, and dorsal to the notopodial chaetae (Figure 8J). The most dorsal column is the first to express *Ct-eve2*. These three columns of *Ct-eve2*-expressing cell clusters span the ectoderm epithelia and have a very similar expression pattern to *elav *[40] and *synaptotagmin *(Meyer and Seaver, unpublished data), conserved markers of neuronal differentiation and of differentiated neurons, respectively. These columns of cell clusters are likely peripheral sensory structures. In addition, at Stages 6 and 7, there is ectodermal expression in a single line of cells across the dorsal surface of some larvae in the PGZ (Figure 8L). This expression domain is not visible at later stages. By Stage 8, there is only weak expression in the columns of ectodermal cell clusters (not shown) and they are undetectable by Stage 9. Expression persists in the hindgut and PGZ into Stage 8 (Figure 8M,O,P), and at this stage it is clear from the subsurface position of the hindgut expression that *Ct-eve2 *is localized to the rectum. A new expression domain appears at Stage 8 in mesodermal cells along the dorsal midline in the posterior mid-body (Figure 8M,N,O). These *Ct-eve2*-expressing cells can be seen extending from the dorsal midline along the anterior edge of segments (Figure 8N). It is unknown to what structures these cells contribute. At Stage 9, expression is limited to a small domain in the rectum (Figure 8Q) and weak expression in the PGZ (Figure 8Q) and dorsal midline (not shown). In juveniles, *Ct-eve2 *is expressed in the PGZ, hindgut and weakly in posterior ganglia (Figure 9B,D).

In summary, *Ct-eve1 *and *Ct-eve2 *have unique spatio-temporal expression patterns, although they are both expressed in mesodermal precursor cells, foregut and hindgut, and the PGZ. Prior to the generation of the mesodermal bands, *Ct-eve1 *and *Ct-eve2 *are similarly expressed in the mesodermal precursor cells (compare Figures 7A and 8A). Once the mesodermal bands appear, however, *Ct-eve1 *is expressed in both mesodermal precursor cells and their descendant bands, whereas *Ct-eve2 *is restricted to the mesodermal precursor cells and is not expressed in their progeny. The temporal dynamics of hindgut expression between the two genes is distinct. *Ct-eve2 *is continuously expressed in the hindgut from Stage 3 through metamorphosis into juvenile stages (Figures 8 and 9). In contrast, *Ct-eve1 *expression is transient in the hindgut, is initially detected a day later than for *Ct-eve2 *at Stage 4, and is not detectable in late stage larvae (compare Figures 7L and 8Q). Another difference between *Ct-eve1 *and *Ct-eve2 *expression is in the mid-body, where *Ct-eve2 *is largely expressed in ectodermal derivatives such as the ventral nerve cord and peripheral sensory structures. This differs dramatically from *Ct-eve1*, which has broad expression throughout the mid-body mesoderm. The unique expression patterns for *Ct-eve1 *and *Ct-eve2 *indicates a divergence of regulatory elements between these two genes.

## Discussion

### *Runt*, *Pax3/7 *and *eve *genes in *C. teleta*

The *C. teleta *genome has clear orthologs of the *D. melanogaster *pair-rule segmentation genes *runt*, *paired *(*Pax3/7*) and *eve*. Two *eve *genes are present in the *C. teleta *genome and, based on amino acid sequence similarity and genomic linkage, they appear to be either the result of a recent duplication or concerted evolution, possibly due to gene conversion event(s) [55,56]. Gene conversion has previously been proposed to occur between the two *Dlx *genes in the *C. teleta *genome [57]. Both *Ct-Pax3/7 *and *Ct-runt *lack a Groucho-binding domain, which is conserved in orthologs of these genes from other species. However, the *Pax3/7A *ortholog in the leech *Helobdella *sp. [58] and the sea squirt *Ciona intestinalis *[59], and the *D. melanogaster paired *gene [60] also lack this motif, as does the predicted limpet *Lottia gigantea runt *homolog. The *C. teleta *genome contains a *groucho *gene (scaffold_692 1858), and other *C. teleta *genes contain the *groucho *repression motif, including the *hes *genes [29]. These features confirm that the *groucho *repression motif is present and likely utilized in the context of transcriptional co-repression in *C. teleta*. Although we do not know the functional consequences of the lack of a groucho domain on the *C. teleta runt *and *paired *homologs, the lack of a groucho domain in *D. melanogaster paired *suggests that this domain is not necessary for a segmentation function.

### Relationship of *Ct-runt*, *Ct-Pax3/7*, *Ct-eve1 *and *Ct-eve2 *expression to segment formation

If annelids and arthropods shared a common segmented ancestor, the expectation is that pair-rule gene orthologs would be expressed in transverse stripes with a consistent relationship to segment boundaries in *C. teleta*, with either a pair-rule or segmental periodicity. However, none of the genes we examined, *Ct-runt*, *Ct-Pax3/7*, *Ct-eve1 *or *Ct-eve2*, have a spatio-temporal expression pattern consistent with a possible role in segment formation. When the first larval segments are forming, *Ct-runt *expression is limited to a subset of cells in the brain and there is no expression in the mid-body where new segments form. Although *Ct-Pax3/7 *is expressed in the mid-body prior to overt segment formation in larvae, its spatial pattern does not prefigure segments, either in a pair-rule or segmental pattern. Both *Ct-eve *genes are expressed in the mid-body as larval segments begin to form. At these stages, the *Ct-eve1 *transcript is ubiquitously expressed in the mid-body mesoderm and lacks spatially restricted positional expression expected for a role segment boundary formation. *Ct-eve2 *expression has more restricted expression in the mid-body, limited to mesodermal precursor cells and the ventral nerve cord, and also lacks pair-rule or segmental stripes of expression in the ectoderm or mesoderm. At late larval stages and during juvenile growth when segments form from the PGZ, all four genes are expressed in the PGZ. However, none of them have patterns that prefigure segments, such as circumferential banded expression or expression limited to either the anterior or posterior region of the growth zone. *Ct-Pax3/7 *and *Ct-runt *have restricted expression around the circumference of the PGZ, in a small ventrolateral domain and ventrolateral domain of the ectoderm, respectively. Both *Ct-eve *genes have broad posterior expression domains that include the PGZ and nascent segments in late larval stages and juveniles, but do not resolve into more restricted patterns. In some animals that form segments sequentially, such as in vertebrates, centipedes and spiders, segment formation is preceded by waves of dynamic gene expression [61-63]. Of the genes examined here, we did not observe dynamic expression in the PGZ, since we did not detect multiple patterns in the PGZ for a gene in similarly staged larvae.

We previously examined expression of orthologs of the pair-rule genes *odd-paired *and *hairy *in *C. teleta *[29,30]. During early stages of segment formation, *Ct-zic*, the *C. teleta *ortholog of *odd-paired*, is expressed along the lateral edge of the ventral neural ectoderm, and in segmentally iterated domains of mesoderm expression in the mid-body soon after segments form. However, there are no stripes of expression in either the ectoderm or mesoderm. One of the *hairy *orthologs, *CapI-hes1*, is expressed in the mesoderm of presumptive larval segments in a segmentally iterated pattern, although it is very distinct from arthropod patterns of *hairy *expression [7,64,65]. Of the pair-rule orthologs for which we have examined the expression pattern in *C. teleta*, *CapI-hes1 *is the most promising candidate to function in segmentation [29]. *Ct-Pax3/7*, Ct*-runt*, *Ct-eve1*, *Ct-eve2*, *CapI-hes2 *and *Ct-zic *all have very distinct expression patterns, all of which indicate that these genes are not likely to be involved in segmentation. Perhaps a subset of these genes are post-transcriptionally regulated and play a role in establishing segmental boundaries; however, functional studies will be necessary to obtain a definitive confirmation of the role of these pair-rule homologs in *C. teleta*.

### Comparison of *runt*, *Pax3/7 *and *eve *expression among species

Expression of the pair-rule genes *Pax3/7 *and *eve *have been characterized in a few other annelids, but expression patterns for *runt *have not been reported for any other lophotrochozoan. *Runt *orthologs appear to have a conserved role in *D. melanogaster *[66] and in *Danio rerio *[67], chick [68] and mammalian neural development (reviewed in [69]), and expression of *Ct-runt *in the brain and ventral nerve cord of *C. teleta *extends this conservation. For *Pax3/7 *orthologs, expression has been examined in two other annelids. Expression of *Pax3/7 *in *P. dumerilii *is somewhat similar to the pattern in *C. teleta *[70]. Both *Pdu-pax3/7 *and *Ct-Pax3/7 *are expressed in bilaterally symmetric longitudinal bands in the ventral trunk ectoderm, adjacent to the ventral nerve cord. In *P. dumerilii*, each band of expression is much broader than in *C. teleta*, possibly reflecting differences in the size of the neurogenic field at these stages. *Helobdella *sp. Austin has two *Pax3/7 *orthologs. The expression pattern has been reported for *Hro-Pax3/7A*, and it is expressed in the mesodermal bands and later in mesodermal derivatives [58]. This is in contrast to the longitudinal bands of *Pax3/7 *expression in the ventral ectoderm in *C. teleta *and *P. dumerilii*. A better understanding of the extent to which these genes have conserved expression domains in annelids will emerge as data from additional species are reported. Outside of annelids, *Pax3/7 *orthologs function in neural specification in the ventral nerve cord of *D. melanogaster *and the vertebrate neural tube. However, their expression patterns are quite distinct in that there are transverse bands of expression in *D. melanogaster *and longitudinal bands of expression in vertebrates [71,72].

Comparison of *eve *expression patterns across bilaterian animals reveals several common expression domains. In annelids, *eve *is expressed in the growth zone during posterior growth of *C. teleta *and *H. robusta*, and in the PGZ during regeneration of *P. dumerilii *[73,74]. However, in *P. dumerilii*, expression in the PGZ is ectodermal, whereas in *H. robusta *and *C. teleta *it is both ectodermal and mesodermal. Furthermore, there is prominent expression in the mesodermal bands of *C. teleta *and *H. robusta*, but not in *P. dumerilii*. Additionally, *eve *is expressed in the posterior gut of both *C. teleta *and *P. dumerilii*. Outside of annelids, posterior expression of *eve *has been reported in *Danio rerio *[75], *Xenopus laevis *[76], *B. floridae *[46], nematodes [77], and in a range of arthropods [10,15,17,78-80]. *Eve *also appears to have an important and widespread role in neural specification or neurogenesis in bilaterians, including in insects (*D. melanogaster *[48,81], *Schistocerca americana *[10]), *Caenorhabditis elegans *[82], *B. floridae *[46], *X. laevis *[83] and *Mus musculus *[84]. In annelids, *eve *is expressed in the developing ventral nerve cord of *C. teleta*, *P. dumerilii *and *H. robusta*, and a role in neural development appears to be the most conserved function of *eve*. Although less well characterized, *eve *has also been implicated in mesodermal patterning in *Danio rerio *[85] and *D. melanogaster *[48]. The expression of *eve *in pericardial progenitors along the dorsal midline in *D. melanogaster *and in mesodermal cells along the dorsal midline of *C. teleta *may indicate a shared role in protostome cardiovascular development. In summary, *eve *genes show conserved roles across bilaterians in multiple domains, indicating conservation of several developmental roles, and the *C. teleta *eve gene expression patterns appear to show conservation with several of these functions. This broad conservation for *eve *is in contrast with its function in arthropod segmentation, which appears to be more of a clade-specific role.

## Conclusions

To the best of our knowledge, no orthologs of *D. melanogaster *pair-rule genes show striped patterns of ectodermal expression in annelids, either in a segmental or pair-rule-like pattern. Vertebrate orthologs of *runt*, *Pax3/7 *and *eve *do not have a role in somitogenesis. Furthermore, a pair-rule-like expression pattern has not been reported for any gene in annelids. In the polychaete *P. dumerilii*, a number of genes are reported to be expressed in segmental ectodermal stripes during larval segment formation, adult segment generation and/or during regeneration. These include members of the *NK *gene family, specifically *NK4*, *Lbx *and *Msx *orthologs [86], the *wnt *genes *Wnt1*, *Wnt4*, *Wnt5*, *Wnt10*, *Wnt11*, *Wnt16 *[24,87], *hh *[88] and *en *[24]. However, with the exception of *en*, *hh*, *Wnt1 *and *Wnt5*, these genes do not function in either arthropod or vertebrate segmentation, and thus are unlikely to be present in an ancestral bilaterian segmentation cascade. Furthermore, there are no functional data implicating any of these genes in the initial formation of segmental boundaries in annelids. Thus, there are likely fundamental differences in how segments form in annelids and arthropods, with no evidence of pair-rule patterning as part of the annelid segmentation program.

## Competing interests

The authors declare that they have no competing interests.

## Authors' contributions

ECS designed the study, performed *in situ *hybridization experiments, did the critical analysis of the data, imaged specimens, prepared figures and wrote the manuscript. GR contributed to gene cloning, generation of riboprobes, *in situ *hybridization experiments and carried out gene orthology analyses. EY carried out gene orthology analyses and contributed to figure preparation and writing of the manuscript. NM carried out staining, confocal microscopy and contributed to figure preparation. All authors contributed to editing of the manuscript and all authors read and approved the final manuscript.

## Supplementary Material

Additional file 1 **Table S1**. List of animal species, gene abbreviations and NCBI accession numbers used for amino acid sequence alignments. Click here for file

Additional file 2 **Figure S1. Ct-Runt groups with Runt protein sequences from other animal taxa**. The tree shown is a Bayesian consensus tree. Posterior probability support is indicated above the nodes, and maximum likelihood bootstrap support values are indicated below the nodes where the two tree topologies agree. Abbreviations are as in the Pax family tree (Figure 2), with the following additional taxa: Amq: *Amphimedon queenslandica*; Bm: *Bombyx mori*; Cs-: *Cupiennius salei*; Fr: *Takifugu rubripes*; Nv: *Nematostella vectensis*; Sk: *Saccoglossus kowalevskii*; Sm: *Schistosoma mansoni*; Sp: *Strongylocentrotus purpuratus*; Tc: *Tribolium castaneum*. Click here for file

Additional file 3 **Figure S2. The two *C. teleta *Eve proteins group together, within a clade that contains Eve sequences from other animals**. The tree shown is a Bayesian consensus tree, with posterior probability support placed above the nodes; maximum likelihood bootstrap support values are placed below the node where the tree topologies agree. Abbreviations are as in the Pax family tree (Figure 2), with the following additional taxa: Aaeg: *Aedes aegyptii*; Ce: *Caenorhabditis elegans*; Hro-: *Helobdella robusta*; Io: *Ilyanassa obsoleta*; Lg: *Lottia gigantea*; Stm-: *Strigamia maritime*; Ttr-: *Theromyzon trizonare*. Click here for file
