# Supplementary material for: Expression of the pair-rule gene homologs runt, Pax3/7, even-skipped-1 and even-skipped-2 during larval and juvenile development of the polychaete annelid Capitella teleta does not support a role in segmentation
Source: EvoDevo. 2012 Apr 18;3:8. doi: 10.1186/2041-9139-3-8 (PMC3359188; doi:10.1186/2041-9139-3-8)
Supplement: Additional file 1 — Table S1. List of animal species, gene abbreviations and NCBI accession numbers used for amino acid sequence alignments. [file 2041-9139-3-8-S1.PDF]

**Gene Abbreviation**	**Species**	**GenBank #**	**Swiss-Prot #**	**JGI Protein #**
Aca-Paxβ	Aplysia californica	DAA12510		
AmPaxA	Acropora millepora	AAC15713		
AmPaxB	Acropora millepora	AAF64460		
AmPaxC	Acropora millepora	AAC15711		
AmPaxD	Acropora millepora	AAF64461		
AmphiPax1	Branchiostoma lanceolatum	CAB42656		
AmphiPax2/5/8	Branchiostoma floridae	O61616		
AmphiPax3/7	Branchiostoma floridae	AAF89581		
AmphiPax-6	Branchiostoma floridae	CAA11367		
Cap-Paxβ	Capitella teleta	DAA12511		
Ci-Pax1/9	Ciona intestinalis	NP_001027594		
Ci-Pax2/5/8	Ciona intestinalis	BAC41498		
Ci-Pax6	Ciona intestinalis	NP_001027641		
**Ct-119889**	**Capitella teleta**			**119889**
**Ct-166874**	**Capitella teleta**			**166874**
**Ct-64892**	**Capitella teleta**			**64892**
**Ct-Pax3/7**	**Capitella teleta**	ABC68267		
**Ct-Pax6**	**Capitella teleta**			**228124**
DjPax-6	Dugesia japonica	BAA75672		
DjPax-6B	Dugesia japonicum	CAC85262.2		
DmEy	Drosophila melanogaster		O18381	
DmGsbd	Drosophila melanogaster		P09082	
DmGsbp	Drosophila melanogaster		P09083	
DmPoxM	Drosophila melanogaster	CAA34860		
DmPoxN	Drosophila melanogaster	CAA41721		
DmPrd	Drosophila melanogaster		P06601	
DmSpark	Drosophila melanogaster	AAB70249		
EsPax6	Euprymna scolopes	AAM74161		
Hau-Pax3/7A	Helobdella sp. Austin	ABI17942.1		
Hau-Pax3/7B	Helobdella sp. Austin	ABL74454.1		
Hau-Pax6A	Helobdella sp. Austin	ABN09915.2		
Hau-Pax6B	Helobdella sp. Austin	ABN09916.2		
Hau-Pax**β**1	Helobdella sp. Austin	ABQ45870.1		
Hau-Pax**β**2	Helobdella sp. Austin	ABQ45871.1		
HsPax-1	Homo sapiens	NP_006183		
HsPax-2	Homo sapiens		Q02962.4	
HsPax-3	Homo sapiens	AAI01301		
HsPax-4	Homo sapiens		O43316	
HsPax-5	Homo sapiens	NP_057953		
HsPax-6	Homo sapiens	NP_000271		
HsPax-7	Homo sapiens	CAA84513		
HsPax-8	Homo sapiens	NP_003457		
HsPax-9	Homo sapiens	NP_006185		
Lgi-Paxβ	Lottia gigantea	DAA12512		
LsPax6	Lineus sanguinus	CAA64847		
MmPax-1	Mus musculus	CAB38370		
MmPax-2	Mus musculus	CAA39302		
MmPax-3	Mus musculus	AAH48699		
MmPax-4	Mus musculus	NP_035168		
MmPax-5	Mus musculus	NP_032808		
MmPax-6	Mus musculus	AAH36957		

MmPax-7	Mus musculus	NP_035169		
MmPax-8	Mus musculus	NP_035170		
MmPax-9	Mus musculus	NP_035171		
Pdu-Pax2/5/8	Platynereis dumerilii	CAD43608		
Pdu-Pax6	Platynereis dumerilii	CAJ40659.1		
SaPby1	Schistocerca americana	AAK82936		
SaPby2	Schistocerca americana	AAK82937		
Sme-Paxβ1	Schmidtea mediterranea	DAA12513		
Sme-Paxβ2	Schmidtea mediterranea	DAA12514		
**Gene Abbreviation**	**Species**	**GenBank #**	**Swiss-Prot #**	**JGI Protein #**
AmqRunx	Amphimedon queenslandica	ACF96957		
BmRunt	Bombyx mori	NP_001104821		
AmphiRunt	Branchiostoma floridae	AAN08567		
**Ct-Runt**	**Capitella teleta**	**ABC68268**		
Cs-Runt-1	Cupiennius salei	CAB89493		
Cs-Runt-2	Cupiennius salei	CAB89494		
DmRunt	Drosophila melanogaster	NP_523424		
DmRunxA	Drosophila melanogaster	NP_001036285		
DmRunxB	Drosophila melanogaster	NP_608399		
MmRunx1	Mus musculus		Q03347	
MmRunx3	Mus musculus		Q64131	
MmRunx2	Mus musculus		Q08775	
NvRunx	Nematostella vectensis	ACF96955		
SkRunx	Saccoglossus kowalevskii	NP_001158429		
SmLozenge	Schistosoma mansoni	XP_002580418		
SpRunt-1	Strongylocentrotus purpuratus	NP_999779		
FrRUNX1	Takifugu rubripes	AAU14191		
FrRUNX2	Takifugu rubripes	AAU14190		
FrRUNX3	Takifugu rubripes	AAU14193		
FrRunt	Takifugu rubripes	NP_001092122		
TcRunt	Tribolium castaneum	EFA09257		
**Gene Abbreviation**	**Species**	**GenBank #**	**Swiss-Prot #**	**JGI Protein #**
AaegEVE	Aedes aegyptii	XP_001652711		
AmphiEvxA	Branchiostoma floridae	XP_002612703		
AmphiEvxB	Branchiostoma floridae	XP_002612702		
CeVab-7	Caenorhabditis elegans		Q93899	
**Ct-Eve1**	**Capitella teleta**	**JQ619657**		
**Ct-Eve2**	**Capitella teleta**	**JQ619658**		
Cs-Eve	Cupiennius salei	CAB89492		
DmEve	Drosophila melanogaster	NP_523670		
Hro-Eve	Helobdella robusta	AAM88672		
Hro-128675	Helobdella robusta			128675
Hro-77445	Helobdella robusta			77445
HsEVX-1	Homo sapiens	NP_001980		
HsEVX-2	Homo sapiens	NP_001073927		
IoEve	Ilyanassa obsoleta	AAO20893		
Lg100793	Lottia gigantea			100793
Lg100789	Lottia gigantea			100789
Pdu-Eve	Platynereis dumerilii	ABA29776		
SkEvx	Saccoglossus kowalevskii	NP_001164694		
SamEve	Schistocerca americana	CAA77869		

SmEve	Schistosoma mansoni	XP_002576083		
StmEve1	Strigamia maritima	ABO77126		
StmEve2	Strigamia maritima	ABO77128		
SpEve	Strongylocentrotus purpuratus	NP_999816		
FrEvx1	Takifugu rubripes	ABF22375		
FrEvx2	Takifugu rubripes	ABF22461		
Ttr-Eve	Theromyzon trizonare	AAL11031		
TcEve	Tribolium castaneum	NP_001034538		
