# Supplementary figures and images for: Expression of the pair-rule gene homologs runt, Pax3/7, even-skipped-1 and even-skipped-2 during larval and juvenile development of the polychaete annelid Capitella teleta does not support a role in segmentation
Source: EvoDevo. 2012 Apr 18;3:8. doi: 10.1186/2041-9139-3-8 (PMC3359188; doi:10.1186/2041-9139-3-8)

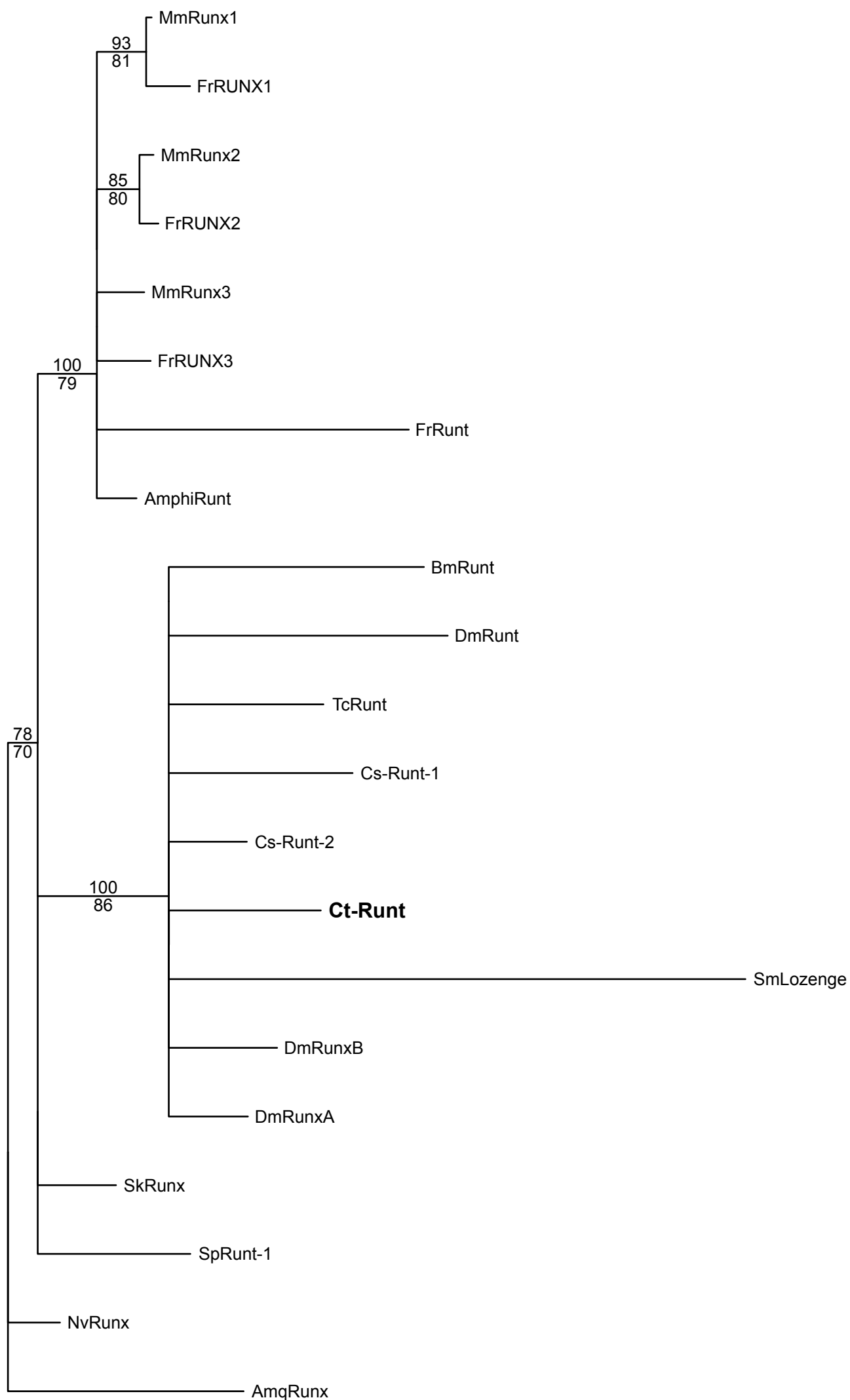

Supplement: Additional file 2 — Figure S1. Ct-Runt groups with Runt protein sequences from other animal taxa. The tree shown is a Bayesian consensus tree. Posterior probability support is indicated above the nodes, and maximum likelihood bootstrap support values are indicated below the nodes where the two tree topologies agree. Abbreviations are as in the Pax family tree (Figure 2), with the following additional taxa: Amq: Amphimedon queenslandica; Bm: Bombyx mori; Cs-: Cupiennius salei; Fr: Takifugu rubripes; Nv: Nematostella vectensis; Sk: Saccoglossus kowalevskii; Sm: Schistosoma mansoni; Sp: Strongylocentrotus purpuratus; Tc: Tribolium castaneum. [file 2041-9139-3-8-S2.PDF]

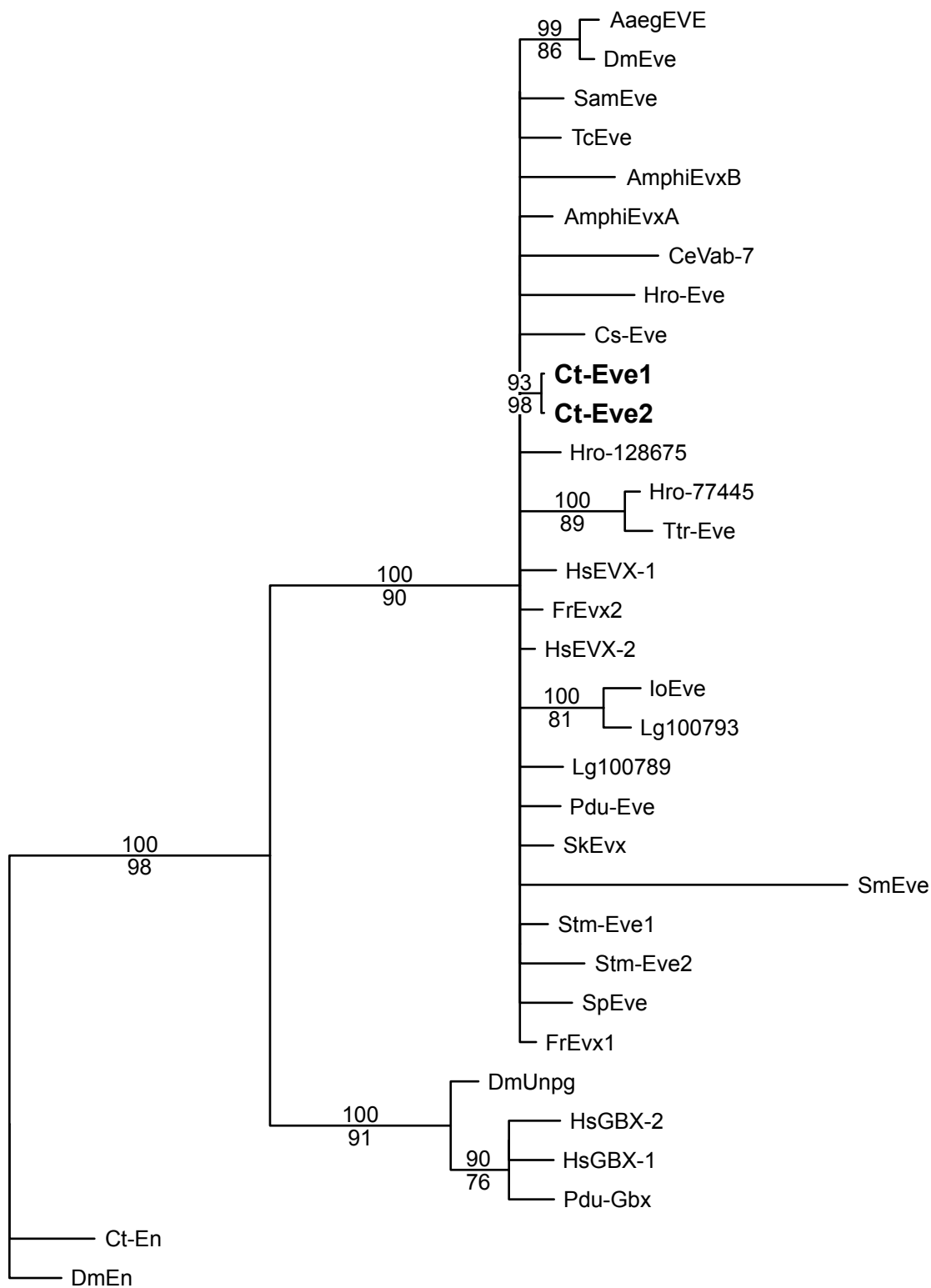

0.3

Supplement: Additional file 3 — Figure S2. The two C. teleta Eve proteins group together, within a clade that contains Eve sequences from other animals. The tree shown is a Bayesian consensus tree, with posterior probability support placed above the nodes; maximum likelihood bootstrap support values are placed below the node where the tree topologies agree. Abbreviations are as in the Pax family tree (Figure 2), with the following additional taxa: Aaeg: Aedes aegyptii; Ce: Caenorhabditis elegans; Hro-: Helobdella robusta; Io: Ilyanassa obsoleta; Lg: Lottia gigantea; Stm-: Strigamia maritime; Ttr-: Theromyzon trizonare. [file 2041-9139-3-8-S3.PDF]
